# Prospectively predicting *Pseudomonas aeruginosa* infection/s using routine data from the UK cystic fibrosis register

**DOI:** 10.1002/hsr2.381

**Published:** 2021-10-01

**Authors:** Nikki Totton, Mike Bradburn, Zhe Hui Hoo, Jen Lewis, Daniel Hind, Carla Girling, Elizabeth Shepherd, Julia Nightingale, Thomas Daniels, Jane Dewar, Sophie Dawson, Mary Carroll, Mark Allenby, Frank Edenborough, Rachael Curley, Charlotte Carolan, Martin Wildman

**Affiliations:** ^1^ Clinical Trials Research Unit, School of Health and Related Research University of Sheffield Sheffield UK; ^2^ Sheffield Adult Cystic Fibrosis Centre Sheffield Teaching Hospital NHS Foundation Trust Sheffield UK; ^3^ Department of Adult Cystic Fibrosis University Hospital Southampton NHS Foundation Trust Southampton UK; ^4^ Wolfson Cystic Fibrosis Centre Nottingham University Hospital NHS Trust Nottingham UK

**Keywords:** adherence, cystic fibrosis, outcome, process, quality improvement

## Abstract

**Rationale and aims:**

Lung health of people with cystic fibrosis (PwCF) can be preserved by daily use of inhaled therapy. Adherence to inhaled therapy, therefore, provides an important process measure to understand the success of care and can be used as a quality indicator. Defining adherence is problematic, however, since the number of prescribed treatments varies considerably between PwCF. The problem is less pronounced among those with *Pseudomonas aeruginosa* (PA), for whom at least three daily doses of nebulized therapy should be prescribed and who thus constitute a more homogeneous group. The UK CF Registry provides routine data on PA status, but data are only available 12 months after collection. In this study, we aim to prospectively identify contemporary PA status from historic registry data.

**Method:**

UK CF Registry data from 2011 to 2015 for PwCF aged ≥16 was used to determine a pragmatic prediction rule for identifying contemporary PA status using historic registry data. Accuracy of three different prediction rules was assessed using the positive predictive value (PPV). The number and proportion of adults predicted to have PA infection were determined overall and per center for the selected prediction rule. Known characteristics linked to PA status were explored to ensure the robustness of the prediction rule.

**Results:**

Having CF Registry defined chronic PA status in the two previous years is the selected definition to predict a patient will have PA infection within the current year (population‐level PPV = 96%‐97%, centre level PPV = 85%‐100%). This approach provides a subset of data between 1852 and 1872 patients overall and a range of 8 to 279 patients per center.

**Conclusion:**

Historic registry data can be used to contemporaneously identify a subgroup of patients with chronic PA. Since this patient group has a narrower treatment schedule, this can facilitate a better benchmarking of adherence across centers.

## 1 INTRODUCTION

Cystic fibrosis (CF) is an archetypal long‐term condition for which there is as yet no cure, but regular use of preventative therapies can improve health outcomes by reducing the frequency of exacerbations and attenuating lung function (forced expiratory volume in 1 second, FEV_1_) decline.^1^ However, adherence to preventative inhaled treatments in CF is generally low (30%‐35%) and low adherence is associated with worse health outcomes.^2^, ^3^ Measuring adherence at an individual level is important for treatment planning and diagnosis in the event of declining lung health. Measuring system‐level adherence is also important because benchmarking across centers within a community of practice can provide a basis to share strategies for improvement. Various quality improvement (QI) initiatives in CF have transformed the delivery of healthcare, for example, streamlined approaches to managing acute exacerbations and increased prescription of efficacious preventative inhaled therapies.^4^ Improving adherence to inhaled therapies is, therefore, a logically important target for QI projects. QI projects focused on adherence can compare between and within centers, therefore providing an understanding of the variation of care across UK centers.

System‐level indicators are most useful when they are independent of local constraints on data capture since the factors that impact quality of care may also impact data capture. Data‐logging nebulizers available in CF can objectively and accurately record each dose of inhaled treatments taken by patients every day.^5^ However, accurate prescriptions data may be unavailable, which impacts the ability to calculate percentage adherence. Yet adherence among people with CF is typically presented as a percentage because the required number of treatment doses will vary according to disease severity. A person without chronic PA and FEV1 80% may only require dornase alfa once daily, whereas someone with chronic PA, FEV1 30% with large volume of sputum may require alternating antibiotics twice daily, dornase alfa once daily, and hypertonic saline twice daily.

Identifying a subgroup of people with CF (PwCF) where the prescription, and therefore denominator for adherence, can be implied using routine data, despite local challenges in data capture, is an important step toward providing adherence comparisons at the center level. One option is to focus on those who have chronic *P. aeruginosa* (PA) infection as consensus exists on the treatment these patients should receive. All major CF guidelines recommend the use of long‐term inhaled antibiotics and mucolytics for people with chronic PA infection, and this provides a minimum denominator of three drugs for those PwCF with chronic PA. Using registry data to identify patients with chronic PA and designating that these patients should receive at least three daily doses of inhaled drugs (denominator) allows adherence data captured by CFHealthHub^6^ (which provides numerator data) to calculate an adherence rate even when prescription data are missing. Although these data will be less accurate in centers without accurate prescription data, this method of calculating adherence data does allow a standardized approach to be used in a well‐defined subset of PwCF allowing center benchmarking that can start an informed exploration of practice.^7^

The aim of system‐level indicators using routine data where possible allows all centers to be included within comparisons, even those that do not have the resources to provide data consistently as the identification of the subgroup with PA uses routine data from the CF Registry. Published UK CF Registry provides historic data on PA status 1 year in arrears. As PA status may fluctuate over time, it is important that the contemporary status is understood as accurately as possible to identify the PA subgroup. Thus, the overarching aim of this paper is to develop a rule to accurately predict which patients will have PA in the current year on the basis of previous years' data collected routinely in the UK CF Registry. It is important to ensure that any prediction rule has an appropriate compromise between sensitivity and specificity to identify a sufficient number of people to allow center comparisons. Thus, the analysis described in this paper sought to determine the optimal rule for prospectively identifying patients who have PA in the current year (trading off accuracy and number). In addition, we explored how other patient characteristics that might be expected to be associated with PA status such as age were related to the PA identifying strategies used.

## 2 METHODS

Retrospective data were collected from the UK CF Registry database for annual reviews between 2011 and 2015 from all UK CF centers. National Health Service (NHS) research ethics approval was granted for the use of UK CF Registry data (Huntingdon Research Ethics Committee 07/Q0104/2), and under these terms, the UK CF Trust Steering Committee approved this study. The data received had pseudo‐anonymized patient and center details.

The following data were obtained from the registry:

- **Demographics**: CF center identifier, gender, age
- **Body mass index** (BMI) in kg/m^2^
- **FEV**_**1**_ during annual review (in % predicted, calculated with GLI equations^8^)
- **Indices of multiple deprivation (IMD)** collected in each of the four UK countries and adjusted to the equivalent English value^9^
- ***P. aeruginosa* status**: chronic (registry definition of three or more positive samples in a 1‐year period, regardless of the number of samples sent), intermittent (one or two positive samples in a 1‐year period), none (no positive samples identified in a 1‐year period)
- **Pancreatic status**: pancreatic insufficient (those on pancreatic replacement therapy [PERT]) or pancreatic sufficient (those not on PERT)
- **CF‐related diabetes (CFRD)**: present or not present, as defined by the UK CF Trust guideline^10^
- **Annual total intravenous (IV) antibiotic use**: total annual number of home and hospital IV antibiotic days
- **Lung transplant**: year of transplant

All adults aged 16 years and above were included in the analyses, with the exception of those undergoing lung transplantation who were excluded from the year of transplant onwards.^11^

For this analysis, we compared the accuracy of three prediction rules that have been selected from clinical knowledge of factors affecting a patient's PA status, and that can be derived from routinely collected data in the CF Registry:

1. Patients with three or more positive PA samples in the previous year (these are classified as “chronic” according to the UK CF Registry guidance),
2. Patients with any positive PA samples in the previous year (“chronic” as above plus patients with one or two PA samples that are classified as “intermittent” according to the UK CF Registry guidance),
3. Patients with any positive PA samples and/or at least 14 days of IV antibiotics (home or hospital) in the previous year.

The rules aim to predict whether a patient will have any positive PA samples within the current year (ie, been classified as “chronic” or “intermittent” by the CF Registry).

The accuracy of the prediction rule was assessed using the positive predictive value (PPV) of the prediction against the outcome of the patients having positive PA samples within the current year based on past years' data. The definition producing the highest PPV (or equivalently, the smallest proportion of patients misclassified as having PA) was taken on to further analysis in which we assessed the impact of increasing the number of years of past data to include in the prediction rule. We calculated the PPV for one, two, three, and four consecutive years' PA diagnosis in order to find the best compromise between accuracy and number of patients included in the subgroup.

Once a final prediction rule had been selected, we used logistic regression to assess the robustness of the prediction rule by calculating the PPV adjusted for other patient characteristics. The covariates included in the model were as follows: age, pancreatic status, FEV_1_, BMI, presence of CFRD, IMD, and IV days all as fixed effects, along with center as a random effect. The functional form of continuous covariates was assessed visually using lowess smoothing^12^ and using fractional polynomials^13^; this is particularly important for age, since the relationship has been shown to be nonlinear within younger patients.^14^

As multiple years' data are available to make predictions, the model was repeated with all data possible, allowing the consistency of the covariates' effect on the prediction to be assessed. The discriminant ability of the model was quantified using the area under the receiver‐operator curve. Since most patients contributed to more than 1 year, the findings of separate predictive models were not combined.

Funnel plots^15^ were created to show the PPV by center and identify any outliers from the prediction rule, using both unadjusted and adjusted PPV. No imputation was performed on missing data. All analyses were performed using R V3.4.1^16^ and Stata V15.^17^

## 3 RESULTS

**FIGURE 1:**
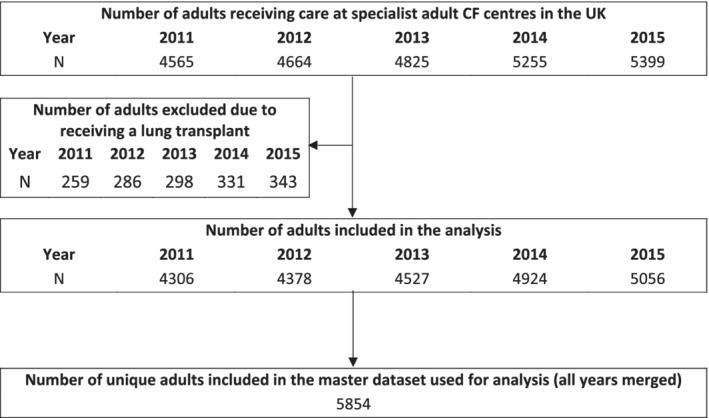
Flowchart of patients within the final dataset

The flowchart in Figure 1 shows the number of patients contributing data to the UK CF Registry in each year. One of the 28 centers did not contribute data to years 2012 and 2013, this center was excluded from all center‐level summaries. The characteristics for the population of patients included in the final master dataset are shown in Table 1, split by year.

**TABLE 1:** Year‐by‐year patient characteristics

	2011	2012	2013	2014	2015
	N = 4306	N = 4378	N = 4527	N = 4924	N = 5056
Age (years)					
Mean (SD)	29.3 (10.1)	29.7 (10.3)	30.0 (10.4)	30.4 (10.6)	30.6 (10.7)
BMI (kg/m^2^)					
Mean (SD)	22.6 (3.8)	22.5 (3.8)	22.6 (3.9)	22.7 (3.9)	22.9 (4.1)
Missing (%)	74 (2%)	136 (3%)	125 (3%)	111 (2%)	124 (2%)
CFRD N (%)					
Yes	1271 (30%)	1342 (31%)	1421 (31%)	1601 (33%)	1676 (33%)
No	3035 (70%)	3036 (69%)	3106 (69%)	3323 (67%)	3380 (67%)
FEV_1_ (% predicted)					
Mean (SD)	62.9 (23.6)	62.0 (23.9)	62.5 (23.8)	63.4 (23.9)	63.4 (23.8)
Missing (%)	110 (2%)	214 (5%)	180 (4%)	200 (4%)	208 (4%)
IV days					
Mean (SD)	25.0 (36.6)	24.7 (38.1)	23.9 (35.8)	23.4 (37.0)	22.0 (35.3)
Missing (%)	58 (1%)	5 (<1%)	5 (<1%)	0 (0%)	0 (0%)
IMD					
Mean (SD)	22.0 (15.7)	21.6 (15.4)	21.7 (15.4)	21.9 (15.6)	22.0 (15.6)
Quintile 1 – least deprived	764 (18%)	795 (18%)	827 (18%)	875 (18%)	898 (18%)
Quintile 2	828 (19%)	853 (19%)	880 (19%)	946 (19%)	981 (19%)
Quintile 3	851 (20%)	875 (20%)	894 (20%)	975 (20%)	999 (20%)
Quintile 4	810 (19%)	822 (19%)	850 (19%)	948 (19%)	956 (19%)
Quintile 5 – most deprived	749 (17%)	735 (17%)	765 (17%)	842 (17%)	879 (17%)
Missing (%)	304 (7%)	298 (7%)	311 (7%)	338 (7%)	343 (7%)
Sex N (%)					
Female	1926 (45%)	1969 (45%)	2053 (45%)	2223 (45%)	2297 (46%)
Male	2380 (55%)	2409 (55%)	2474 (55%)	2701 (55%)	2759 (54%)
*P. aeruginosa* status N (%)					
Chronic	2447 (57%)	2457 (56%)	2428 (54%)	2477 (50%)	2432 (48%)
Intermittent	532 (13%)	527 (12%)	605 (13%)	726 (15%)	709 (14%)
None	1327 (31%)	1394 (32%)	1494 (33%)	1721 (35%)	1915 (38%)
Pancreatic status N (%)					
Insufficient	3508 (81%)	3574 (82%)	3682 (81%)	4008 (81%)	4107 (81%)
Sufficient	699 (16%)	727 (17%)	774 (17%)	869 (18%)	886 (18%)
Missing	99 (2%)	77 (2%)	71 (2%)	47 (1%)	63 (1%)

Abbreviations: BMI, body mass index; CFRD, cystic fibrosis related diabetes; FEV_1_, forced expiratory volume; IMD, indices of multiple deprivation; IV, intravenous.

For each of the four time periods, 94% of patients with chronic PA were found to have PA in the subsequent year. The other prediction rules included a greater number of patients but a smaller proportion with PA (range 79%‐90%; Table 2).

**TABLE 2:** PPV for three initial prediction rules

Prediction rule	Predicted year; PPV (%)			
	2012 N = 3931	2013 N = 4093	2014 N = 4284	2015 N = 4630
Chronic PA status	94% (93%, 95%)	94% (93%, 95%)	94% (93%, 95%)	94% (94%, 95%)
Chronic or intermittent PA status	90% (89%, 91%)	90% (89%, 91%)	88% (87%, 89%)	87% (86%, 88%)
Chronic or intermittent PA status or ≥ 14 IV days	84% (83%, 86%)	83% (82%, 85%)	81% (79%, 82%)	79% (78%, 81%)

Abbreviations: IV, intravenous; PA, *Pseudomonas aeruginosa*; PPV, positive predictive value.

**FIGURE 2:**
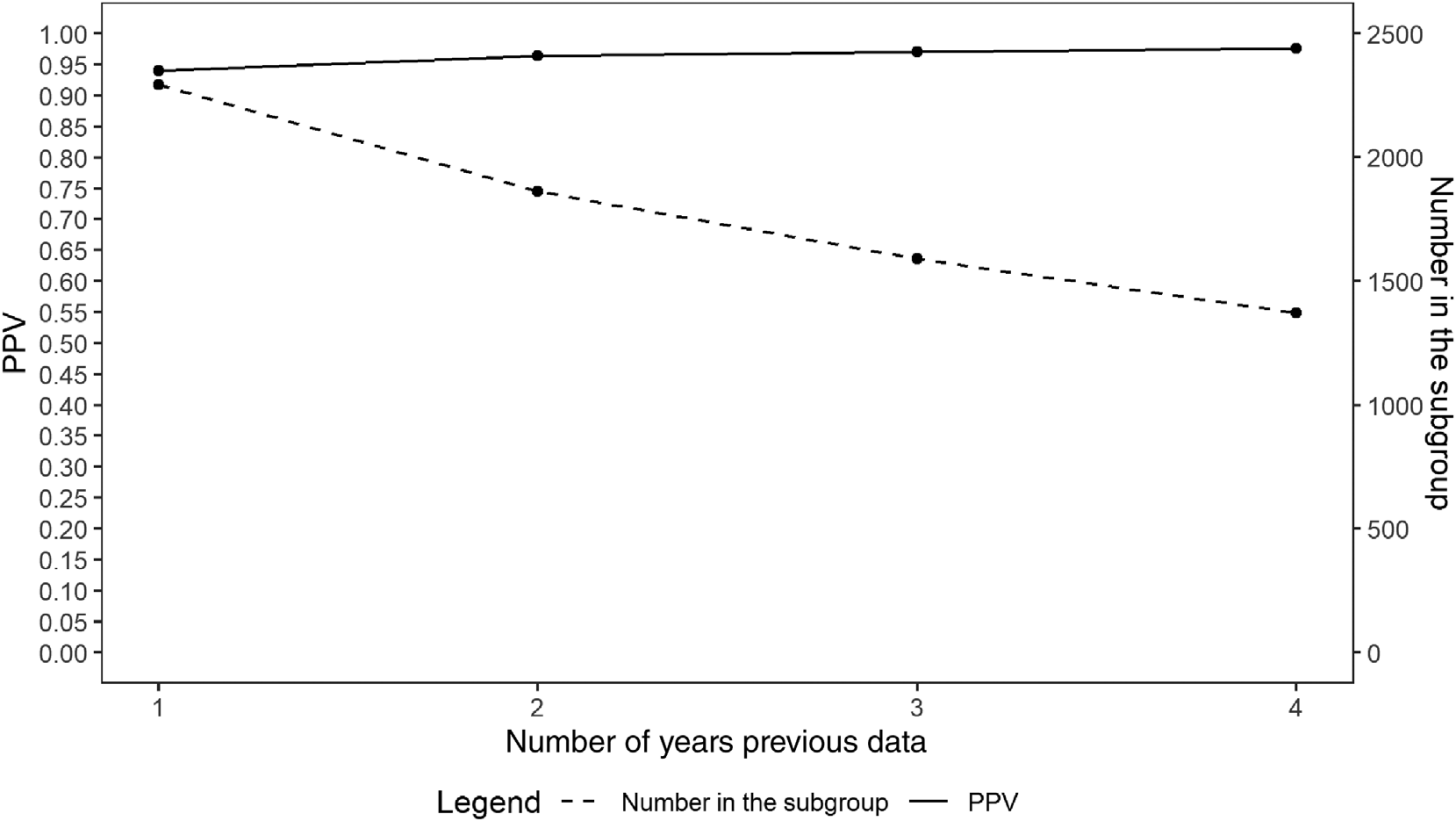
Trade‐off between PPV and number within the subset for each number of years' data (averaged across all years)

**FIGURE A1:**
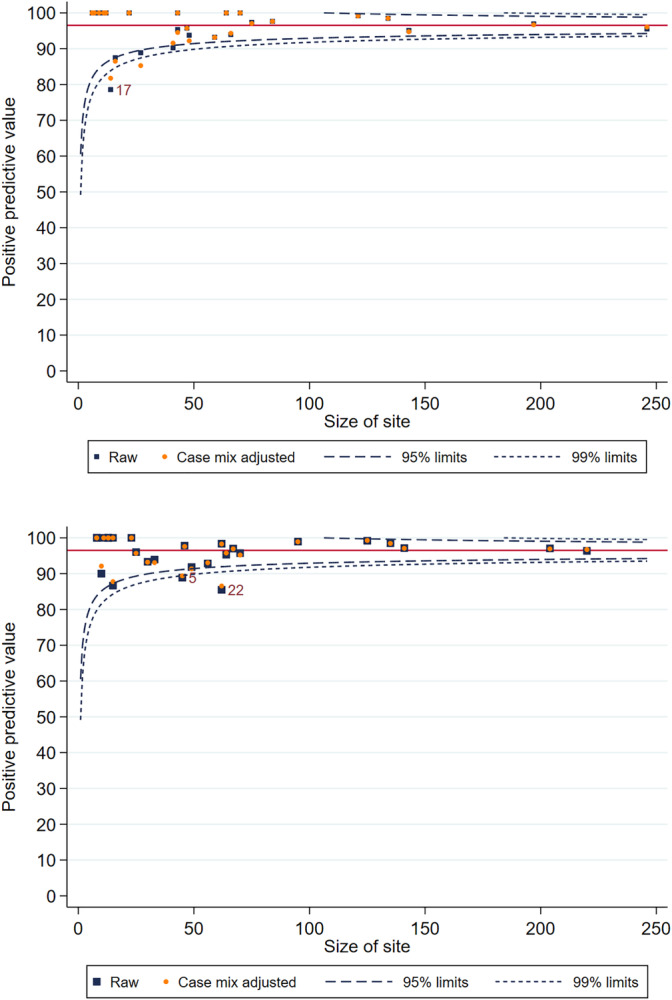
Funnel plot for adjusted and unadjusted PPV by center for 2014 (top) and 2015 (bottom) predictions, lines reflect 95% (inner) and 99% (outer) reference intervals

Using the prediction rule of chronic PA status in previous years, the optimum number of consecutive years' data to use was explored and the results are shown in Table 3. As expected, the PPV increases with extra years' data (range: 94%‐98%), and Figure 2 shows this against the number within the subset, which decreases as more years' data are included. Taking the two together suggests the optimal threshold for the prediction rule is to select those with two successive years of a chronic PA status to predict the current years' status, thereby allowing a sufficiently large sample to be used for future research (average N = 1862) without appreciably reducing the confidence in the accuracy of the diagnosis (average PPV = 96%). The contingency table for the PPV calculations of this chosen prediction rule for 2013 is shown in Table 4, and the contingency tables for 2014 and 2015 are in Table A1 and the resulting funnel plots in Figure A1.

**TABLE 3:** Positive predictive value (PPV) for four different years' data in the prediction rule

Number of consecutive previous years	Predicted year; PPV (%)			
	2012	2013	2014	2015
1 year	94% (93%, 95%)	94% (93%, 95%)	94% (93%, 95%)	94% (94%, 95%)
2 years	‐	97% (96%, 97%)	96% (96%, 97%)	96% (95%, 97%)
3 years	‐	‐	97% (96%, 98%)	97% (96%, 98%)
4 years	‐	‐	‐	98% (97%, 98%)

**TABLE 4:** Contingency table comparing the definition of two previous years' chronic *Pseudomonas aeruginosa* (PA) status (2011 and 2012) to predict a patient having positive PA samples within the current year (2013) compared with a confirmed positive PA sample in that year (2013)

Prediction year = 2013		Outcome		
		Positive samples	No positive samples	Total
Prediction	Positive samples	1801 (97%)	60 (3%)	1861
	No positive samples	811 (44%)	1038 (56%)	1849
	Total	2612	1098	3710

### 3.1 Center comparisons

The PPV was reasonably consistent across the 27 centers (range: 85%‐100%). The number selected in the subgroup for each center had a median of at least 49 (from 2013 prediction year) with a range from 8 to 279 (over all prediction years). More details are in Table A2.

### 3.2 Predictive modeling

The final analyses sought to identify whether the PPV were associated with the patient's demographic and clinical features. These analyses included patients that were recorded as having PA in two successive years and were aged under 60. Patients over 60 are both uncommon and also much more likely to be pancreatic sufficient than other age groups, suggesting that they have a preponderance of less severe phenotypes. Analyses were undertaken separately for patients in 2013, 2014, and 2015 separately (Table 5).

**TABLE 5:** Regression model output using the outcome of a patient having positive PA samples within a given year and patient characteristics as predictors

Variable	Model 1 (2013)	Model 2 (2014)	Model 3 (2015)
	Coefficient (SE)		
Age	a *	a*	a
BMI	−0.013 (0.047)	−0.075 (0.039)	−0.033 (0.042)
CF‐related diabetes	0.730 (0.373)	0.717 (0.337)*	−0.267 (0.283)
FEV_1_ (% pred)	−0.279 (0.737)	−0.888 (0.714)	−0.219 (0.705)
IV days	0.006 (0.005)	0.002 (0.005)	0.012 (0.006)
Pancreatic insufficiency	0.365 (0.446)	0.017 (0.426)	0.841 (0.390)*
Female sex	0.017 (0.296)	0.450 (0.275)	0.062 (0.270)
IMD	0.009 (0.010)	0.002 (0.009)	0.010 (0.009)
Area under ROC curve	0.664	0.678	0.628

*Note*: *P < .05; **P < .01.

Abbreviations: BMI, body mass index; CF, cystic fibrosis; IMD, indices of multiple deprivation; IV, intravenous; PA, *Pseudomonas aeruginosa*.

^a^ Age was nonlinear with different functional forms: its effect on PPV plateaued after the age of 30 in all cases.

The models for three different years showed no consistency for significant predictors, and only age appeared more than once. In all years, the predictive ability of the model was low with an area under the curve of 0.66, 0.68, and 0.63, respectively, suggesting a small influence of the variables to the prediction. This suggests that the chosen rule of “chronic” PA for two successive years without an adjustment for patient characteristics is sufficient.

**FIGURE 3:**
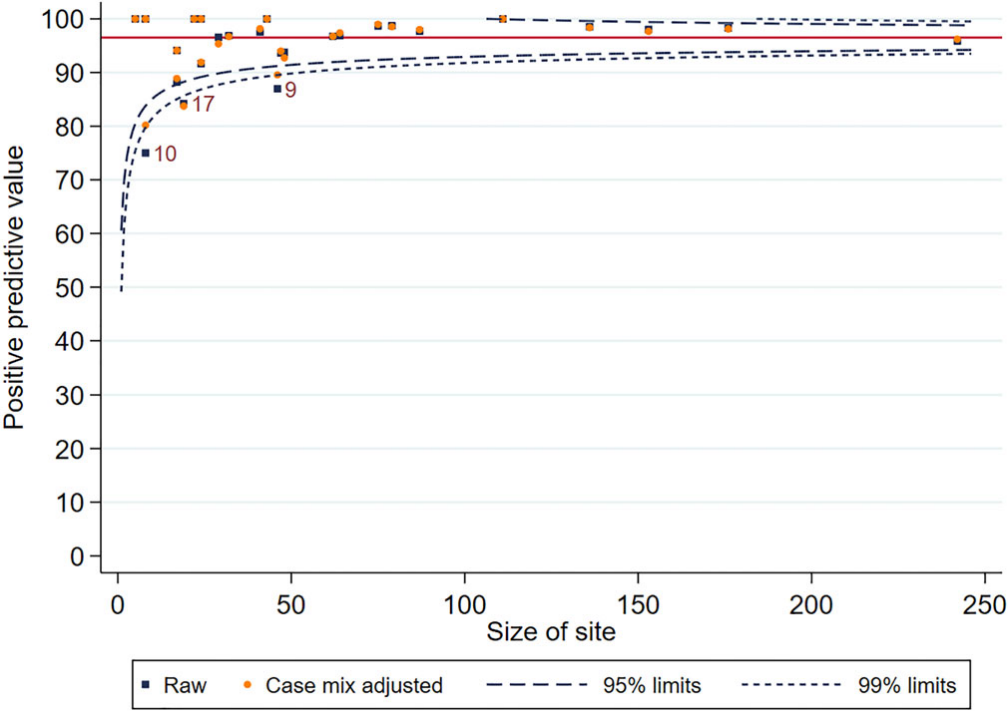
Funnel plot for adjusted and unadjusted PPV by center for 2013 predictions, lines reflect 95% (inner) and 99% (outer) reference intervals

Furthermore, the adjusted PPV at center level is very similar to the unadjusted PPV, as shown in the funnel plot for 2015 (Figure 3). The difference in the precision of the prediction is minimal, giving a strong rationale that adjusting for covariates does not substantially affect the accuracy of the predictions.

## 4 DISCUSSION

Patients with positive PA samples can be accurately identified in advance using routine data and a simple clinical rule. Patients with a chronic PA status reported within the UK CF Registry for the previous two consecutive years are likely to remain positive in the subsequent year, with minimal variation between patients of differing presenting characteristics or across treatment centers.

There are limitations to this work due to the data architecture of the UK CF Registry. Firstly, if PA status is reported as “none,” this could be because a person did not have PA infection, but alternatively may arise by the patient not producing any sputum at clinic visits or by missing data. Secondly, advances in CF treatments, including treatments for PA infection,^18^, ^19^, ^20^ means that the definition in this study should be re‐explored again with future registry data. Thirdly, although guidance on the definition of chronic and intermittent *P. aeruginosa* status is provided by the CF Registry, it is uncertain how closely this definition is followed by individual CF centers. As encounter‐based data are not available for the data CF Registry dataset that was used, confirmation was not possible, and so an important next step will be to validate this rule against a gold standard definition such as the Leeds criteria,^21^ a formally elicited consultant judgment or a consensus definition.

Nevertheless, this prediction rule appears robust to differing patient characteristics and may allow center comparisons to be carried out using registry data. This is important, since center comparisons underpin important advances in CF. For example, the comparisons of CF centers in Toronto and Boston in the 1980s identifying the importance of aggressive nutritional support^22^ is now a standard of care in CF.^23^

Due to the low prevalence of CF and the high numbers of patients required to make comparisons based on key outcomes such as FEV_1_,^24^ process measures such as adherence are important in providing sensitive measures to identify differences in quality of care^25^ with higher adherence to treatments linked to better health outcomes.^26^ The subgroup of people predicted to have PA in the current year based on historic registry data provide a homogenous group in whom to compare adherence rates with consensus about minimum treatment regimens (mucolytic plus antibiotic), allowing a minimum denominator to be imputed if prescription data are unavailable^27^, ^28^, ^29^).

Additionally, the use of convenience samples is a well‐understood limitation of comparing adherence between cohorts or centers.^30^ By using this prediction rule to identify patients from a registry dataset (more than 95% complete), researchers can understand to what extent reported adherence data in a center has included all patients with chronic PA. Identification of the number of patients included in adherence rates within a center, compared with the numbers in the historic registry data, allows an understanding of how many patients are missing, thus illuminating the potential impact of sampling error on estimates of center‐level adherence. This work supports the national indicator for CF patients^31^ that has been developed to use adherence data from people with chronic PA to assess center‐level adherence and establish the missing patients within the center.

## 5 CONCLUSION

The routine use of data‐logging nebulizers in CF allows the number of nebulizer doses used to be captured accurately at an individual level, but there is uncertainty regarding the method to robustly compare system‐level adherence between different CF centers. The identification of a subgroup of adults predicted to have PA infection using routine data provides a first step toward destination robust comparison of system‐level adherence between centers since there is consensus regarding treatment targets in this subgroup. We have developed a method of using routine data within the UK CF Registry to identify a subgroup of adults who are predicted, with good accuracy, to have a PA infection in the given year. The next step is to validate our method against other definitions of chronic PA infection.

## FUNDING

This output is part of the CFHealthHub Data Observatory study, which is funded by NHS England Commissioning for Quality and Innovation.

## CONFLICT OF INTEREST

The authors declare that they have no conflicts of interest.

## TRANSPARENCY STATEMENT

The authors confirm this manuscript is an honest, accurate, and transparent account of the study being reported; that no important aspects of the study have been omitted; and that any discrepancies from the study as planned (and, if relevant, registered) have been explained.

## AUTHORS' CONTRIBUTIONS

Conceptualization: Jen Lewis, Zhe Hui Hoo, Daniel Hind, Carla Girling, Martin Wildman, Elizabeth Shepherd, Julia Nightingale, Thomas Daniels, Jane Dewar, Sophie Dawson, Mary Carroll, Mark Allenby, Frank Edenborough, Rachael Curley, Charlotte Carolan.

Data Curation: Nikki Totton, Mike Bradburn, Jen Lewis, Zhe Hui Hoo, Carla Girling.

Formal Analysis: Nikki Totton, Mike Bradburn, Jen Lewis.

Funding Acquisition: Daniel Hind, Martin Wildman.

Methodology: Nikki Totton, Mike Bradburn, Jen Lewis, Zhe Hui Hoo, Martin Wildman.

Project Administration: Carla Girling.

Validation: Mike Bradburn.

Visualization: Nikki Totton, Mike Bradburn, Jen Lewis.

Writing—Original Draft Preparation: Nikki Totton, Zhe Hui Hoo, Daniel Hind, Martin Wildman.

Writing—Review and Editing: Zhe Hui Hoo, Mike Bradburn, Jen Lewis, Carla Girling, Elizabeth Shepherd, Julia Nightingale, Thomas Daniels, Jane Dewar, Sophie Dawson, Mary Carroll, Mark Allenby, Frank Edenborough, Rachael Curley, Charlotte Carolan.

Martin Wildman had full access to all of the data in the study and takes complete responsibility for the integrity of the data and the accuracy of the data analysis.

## CONSENT TO PARTICIPATE

De‐identified data on adults with cystic fibrosis was requested and obtained from the CF Registry in accordance with ethical approval.

## ETHICS STATEMENT

National Health Service (NHS) research ethics approval was granted for the use of UK CF Registry data (Huntingdon Research Ethics Committee 07/Q0104/2) and under these terms, the UK CF Trust Steering Committee approved this study.

## Data Availability

The datasets used and/or analyzed during the current study are available from the corresponding author on reasonable request subject to CF Registry approval.

## Appendix

**TABLE A1:** Contingency table comparing the definition of two previous years' chronic PA status to predict a patient having positive PA samples within the current year (2014 and 2015 respectively) compared with a confirmed positive PA sample in that year (2014 and 2015 respectively)

Prediction year = 2014		Outcome		
		Positive samples	No positive samples	Total
Prediction	Positive samples	1804 (96%)	68 (4%)	1872
	No positive samples	826 (41%)	1189 (59%)	2015
	Total	2630	1257	3887

Prediction year = 2015		Outcome		
		Positive samples	No positive samples	Total
Prediction	Positive samples	1782 (96%)	70 (4%)	1852
	No positive samples	859 (39%)	1333 (61%)	2192
	Total	2641	1403	4044

**TABLE A2:** Summary of CF center‐level PPV, the number and proportion of patients that are predicted to have positive PA samples in the given year

	Prediction year		
	2013	2014	2015
PPV			
Median	98%	97%	97%
Min	85%	86%	86%
Max	100%	100%	100%
IQR	94%‐99%	94%‐100%	94%‐99%
Number predicted to have positive PA samples			
Median	49	53	52
Min	8	10	9
Max	273	279	249
IQR	27‐82.5	22‐82.5	19.5‐76
Proportion predicted to have positive PA samples			
Median	40%	37%	37%
Min	13%	8%	10%
Max	53%	55%	55%
IQR	32%‐46%	31%‐46%	28%‐44%

Abbreviations: CF, cystic fibrosis; PA, *Pseudomonas aeruginosa*; PPV, positive predictive value.
